# Experimental data for the magnetic properties of vulcanized natural rubber nanocomposites using vibrating sample magnetometer (VSM)

**DOI:** 10.1016/j.dib.2022.108872

**Published:** 2023-01-04

**Authors:** Rozaina Ismail, Azmi Ibrahim, Hamidah Mohd.Saman@Hj. Mohamed, Mohamad Rusop Mahmood, Azlan Adnan

**Affiliations:** a School of Civil Engineering, College of Engineering, Universiti Teknologi MARA, Shah Alam, Selangor 40450, Malaysia; b School of Electrical Engineering, College of Engineering, Universiti Teknologi MARA, Shah Alam, Selangor 40450, Malaysia; c Faculty of Civil Engineering, Universiti Teknologi Malaysia, Skudai, Johor 81318, Malaysia

**Keywords:** Magnetic properties, Carbon nanotubes, Microcarbonyl iron, VSM test, Nanocomposite, Elastomer, Natural rubber, Rubber bearing, VSM, Vibrating Sample Magnetometer, MCI, Microcarbonyl iron, SMR, Standard Malaysian Rubber, ZnO, zinc oxide, CBS, Cyclohexyl Benzothiazolesulfenamide, TMTD, Tetramethylthiuram Disulphide, MWCNT, Multi-Walled Carbon Nanotube, BS, British Standard, phhr, per hundredrubber, UiTM, Universiti Teknologi MARA, ReNeU, Research Nexus UiTM, InQKA, Institute of Quality and Knowledge Advancement

## Abstract

Base isolation is a technique installed to absorb any movement or vibration on the structures. The incorporation of nanocomposites into elastomer as the interesting materials especially for the active stiffness and vibration control of structural systems. A base isolator is made up of alternate layers of steel and rubber. The performance of magnetic rubber device is dependent on mechanical and magnetic properties of composite rubber materials. A vibrating sample magnetometer (VSM) is an instrument to detect the magnetic properties. The article provides information on the magnetic properties corresponding to different carbon nanotubes loadings of 0%, 1%, 3% and 5% and different loading of microcarbonyl iron (MCI) i.e. 0% (B0), 10% (B10), 20% (B20) and 30% (B30) in natural rubber compound. The magnetic properties dataset described the data from compression test.

**Specifications Table**

**Table:** 

Subject	Civil and Structural Engineering, Composite, Material Science Engineering
Specific subject area	Material Characterization, Mechanical Properties, Polymer Physics.
Type of data	Table Text Graph Figure
How the data were acquired	Vibrating Sample Magnetometer (VSM) Lakeshore 7404 Series
Data format	Raw Analyzed
Description of data collection	The cure characteristic parameters measured include scorch time, cure time, maximum torque and torque differences. The compound batches had been left at least 16 h before being cut and tested. The curing temperature was conducted at 150 °C. The test pieces had been conditioned at 23 °C for at least three hours before conducting the testing. The composite samples were measured at room temperature in the range of approximately –15,000 to +15,000 Gauss (G) magnetic field with magnetic moment of 3 to 5 emu/g. The samples were compounded under two (2) variations; i) containing the same amount of magnetic fillers, which is about 30 pphr in the composites but vary in nanocarbon loading i.e. 0%, 1%, 3% and 5% and ii) samples containing the same amount of 1 pphr nanocarbon fillers, in the composites with different loading of microcarbonyl iron (MCI) i.e. 0% (B0), 10% (B10), 20% (B20) and 30% (B30). Five test pieces of samples were prepared in the range of 2 mm x 2 mm to 3 mm x 3 mm, as small as possible to suit the 4 mm x 4 mm tube holder of the VSM to investigate the magnetic properties of vulcanized natural rubber nanocomposites.
Data source location	Data obtained from the paid laboratory, Center for Applied Physics Study, Faculty of Science and Technology, Universiti Kebangsaan Malaysia.
Data accessibility	Repository name: Mendeley Data Data identification number: 10.17632/2dytmtksnc.1 Direct URL to data: https://data.mendeley.com/datasets/2dytmtksnc

## Value of the Data

• A vibrating-sample magnetometer (VSM) test is a scientific instrument that measures magnetic properties of materials as a function of magnetic field to find deposits of iron because they can measure the magnetic field variations caused by the deposits. The precision and accuracy of VSM's are quite high even among other magnetometers and VSM's further allow for a sample to be tested at varying angles with respect to its magnetization letting researchers minimize the effects of external influences.
• The 2 sets of data presented shows that set 1; the four different carbon nanotubes loadings of 0%, 1%, 3% and 5% and set 2; four different loading of microcarbonyl iron with 0%, 10%, 20% and 30%. Hence, this data is useful because it includes a variety of filler types and tested with different sample percentages. The choice of the different carbon nanotubes loading and microcarbonyl iron loading used in this study due to the most optimum values adopted by many researchers for the application of carbon nanotubes and microcarbonyl iron for elastomer.
• Data presented here could be helpful in further research on magnetic rubber modification of natural rubber compound. Due to rubber properties are depending on compounding ingredients especially vulcanization system, type and amount of filler and other special ingredients for better performance.
• These data have important significance for the basic parameters for the design of elastomeric bearings used for isolation of structure from external vibration like earthquake.

## 1 Objective

I To characterize and enhance the mechanical properties of nanocomposite natural rubber elastomer.
II To determine magnetic properties of nanocomposite material using Vibrating Sample Magnetometer (VSM) Test.

## 2 Data Description

Data presented in this article was used to investigate the performance of nanocomposites elastomer due to effect of carbon nanotubes loading. The mechanical test is conducted through this magnetic test which affect the dispersion of the fillers hence. The data are focused on the mechanical properties of magnetic iron filled natural rubber composites which gives significant effect to the magnetic properties of sample to be used as elastomer for Seismic Isolator.

The raw data of the magnetic test for with different nanocarbon loading are tabulated in Table 1 and the raw data for the Effect of variation of microcarbonyl iron are tabulated in Table 2.

**Table 1:** Raw data for the magnetic properties of samples with different nanocarbon loading.

	Moment/Mass (emu/g)				
Field (G)	MCI	A0	A1	A3	A5
14,000	0.200246	0.0283913	0.011856	1.69319	1.4611
13,000	0.200059	0.0284546	0.011935	1.69	1.45872
12,000	0.199789	0.0285044	0.012014	1.68612	1.45573
11,000	0.199427	0.0285393	0.012087	1.6809	1.4521
9999.99	0.198915	0.0285592	0.012154	1.67431	1.4474
9000	0.198296	0.0285611	0.012208	1.66548	1.44123
8000	0.197443	0.0285389	0.012258	1.65287	1.43247
7000.01	0.196237	0.0284836	0.012286	1.63283	1.41803
6000	0.194458	0.0283335	0.012252	1.57742	1.37479
4999.99	0.191411	0.0279222	0.012053	1.42852	1.2533
4000	0.185754	0.0269847	0.011569	1.20998	1.07395
3000	0.17435	0.024999	0.010633	0.945014	0.853495
2000	0.150288	0.0210464	0.008896	0.648157	0.598785
999.993	0.101002	0.01365	0.005783	0.328781	0.312152
949.995	0.0974672	0.0131525	0.005576	0.310656	0.296309
899.997	0.0938717	0.0126379	0.005363	0.293287	0.280774
849.995	0.090144	0.0121116	0.005144	0.276302	0.265311
799.997	0.086284	0.0115662	0.004919	0.259489	0.249905
750	0.0822864	0.0110037	0.004688	0.242874	0.234479
699.994	0.0781325	0.0104254	0.00445	0.226413	0.21903
650	0.0738305	0.00982728	0.004202	0.210024	0.203579
599.996	0.0693508	0.00921033	0.003949	0.193705	0.188137
549.997	0.0646792	0.00857176	0.003686	0.17747	0.172626
499.995	0.0598191	0.00791101	0.003415	0.161275	0.157094
449.995	0.0547479	0.00722631	0.003135	0.145147	0.141598
399.996	0.0494411	0.00651755	0.002843	0.129032	0.126023
349.974	0.0440771	0.00580669	0.002549	0.113376	0.110835
300	0.0383512	0.0050519	0.00224	0.097402	0.095307
249.996	0.0323635	0.00426546	0.001917	0.081394	0.079707
199.997	0.0261719	0.0034592	0.001582	0.065418	0.064132
149.999	0.019832	0.00263215	0.001237	0.049471	0.04854
100.002	0.0134285	0.00179207	0.00088	0.033565	0.03296
50.0003	0.00705612	0.000946211	0.000503	0.017682	0.017377
−0.00434681	0.000756963	0.000101451	9.44E-05	0.001816	0.001755
−50.0067	−0.00543174	−0.000738799	−0.00033	−0.01405	−0.01388
−99.9987	−0.0115926	−0.00157532	−0.00072	−0.02989	−0.02948
−150.004	−0.017742	−0.00240564	−0.00109	−0.04572	−0.04505
−199.997	−0.0238075	−0.00322358	−0.00145	−0.06154	−0.06061
−250	−0.0297401	−0.0040227	−0.00179	−0.07737	−0.07617
−300.005	−0.0354953	−0.00479983	−0.00213	−0.09314	−0.0917
−350.003	−0.0410576	−0.00555427	−0.00245	−0.10892	−0.10721
−400.007	−0.0463795	−0.00627037	−0.00274	−0.12435	−0.12235
−450.005	−0.0515724	−0.00697653	−0.00303	−0.1401	−0.13776
−500.005	−0.0565764	−0.00765936	−0.00332	−0.15585	−0.15319
−550	−0.0614078	−0.0083209	−0.00359	−0.17159	−0.16856
−599.999	−0.0660517	−0.00896045	−0.00386	−0.18731	−0.18388
−650	−0.0705313	−0.0095799	−0.00411	−0.20301	−0.19917
−700.004	−0.0748544	−0.0101785	−0.00436	−0.21871	−0.21441
−750.001	−0.079022	−0.0107628	−0.0046	−0.2344	−0.22958
−800.003	−0.0830434	−0.0113261	−0.00484	−0.25004	−0.2447
−850.003	−0.0869283	−0.011874	−0.00506	−0.26567	−0.25973
−900.005	−0.090675	−0.0124044	−0.00528	−0.28131	−0.27468
−950.001	−0.0942988	−0.0129203	−0.00549	−0.29692	−0.28965
−1000	−0.097806	−0.0134216	−0.0057	−0.31251	−0.30451
−2000.01	−0.147953	−0.0209158	−0.00883	−0.61824	−0.5875
−3000.02	−0.172623	−0.0249309	−0.01058	−0.90845	−0.84047
−4000.01	−0.184485	−0.0269436	−0.01153	−1.17611	−1.06179
−5000.01	−0.190504	−0.0279007	−0.01202	−1.40616	−1.24417
−6000.02	−0.193829	−0.0283156	−0.01223	−1.56735	−1.36876
−7000	−0.19587	−0.0284713	−0.01226	−1.62956	−1.41425
−8000.01	−0.197192	−0.0285335	−0.01223	−1.65147	−1.42845
−9000.01	−0.198126	−0.0285514	−0.01218	−1.66438	−1.43703
−10,000	−0.198813	−0.0285477	−0.01213	−1.67341	−1.44276
−11,000	−0.199338	−0.0285299	−0.01206	−1.68012	−1.44723
−12,000	−0.199748	−0.0284965	−0.01199	−1.68564	−1.45073
−13,000	−0.200032	−0.0284511	−0.01191	−1.68972	−1.45319
−14,000	−0.200241	−0.0283963	−0.01183	−1.69293	−1.45498
−13,000	−0.200057	−0.0284565	−0.01191	−1.69008	−1.45314
−12,000	−0.19976	−0.0285016	−0.012	−1.68615	−1.45051
−11,000	−0.199385	−0.0285417	−0.01206	−1.68114	−1.44724
−10,000	−0.198906	−0.0285583	−0.01213	−1.67485	−1.44285
−9000	−0.198289	−0.0285632	−0.01219	−1.66689	−1.43681
−8000	−0.197456	−0.0285482	−0.01224	−1.65511	−1.42856
−7001.32	−0.196313	−0.0284879	−0.01227	−1.63686	−1.41498
−6000	−0.194565	−0.0283452	−0.01224	−1.58488	−1.37301
−4999.99	−0.191645	−0.0279377	−0.01204	−1.44387	−1.25461
−4000	−0.186145	−0.027013	−0.01157	−1.23016	−1.07717
−2999.99	−0.174956	−0.0250674	−0.01064	−0.96673	−0.85765
−2000	−0.151194	−0.0211414	−0.00892	−0.66658	−0.60281
−999.994	−0.102016	−0.0137482	−0.00581	−0.33928	−0.31444
−949.998	−0.0984808	−0.0132455	−0.0056	−0.3203	−0.29843
−899.999	−0.0948678	−0.0127299	−0.00539	−0.30217	−0.28276
−849.999	−0.091148	−0.0122003	−0.00517	−0.28441	−0.26714
−799.998	−0.0872762	−0.0116535	−0.00494	−0.26699	−0.25154
−750	−0.0832797	−0.011091	−0.00471	−0.24976	−0.236
−699.998	−0.0790995	−0.0105074	−0.00447	−0.23261	−0.22041
−649.997	−0.0747716	−0.00990653	−0.00422	−0.21562	−0.20483
−600.002	−0.0702664	−0.00928419	−0.00397	−0.19873	−0.18921
−550	−0.0655736	−0.00864226	−0.0037	−0.18198	−0.1736
−500	−0.0606693	−0.00797503	−0.00343	−0.16523	−0.15793
−450.002	−0.0555649	−0.0072854	−0.00315	−0.14855	−0.14226
−399.996	−0.0502297	−0.00657226	−0.00286	−0.13201	−0.12662
−349.998	−0.0447031	−0.00584306	−0.00256	−0.11576	−0.11122
−300.002	−0.038888	−0.00507888	−0.00225	−0.09934	−0.09557
−250.004	−0.0328256	−0.00428812	−0.00192	−0.08291	−0.07987
−200.001	−0.0265304	−0.00347225	−0.00158	−0.06651	−0.06415
−150.004	−0.0200616	−0.00263598	−0.00123	−0.05015	−0.04845
−99.9968	−0.0135065	−0.00178424	−0.00087	−0.03384	−0.03275
−49.9977	−0.0069613	−0.000926786	−0.0005	−0.01757	−0.01707
0.00177898	−0.00049	−6.96225E-05	−7.4E-05	−0.00134	−0.00134
49.995	0.00591506	0.000783023	0.000357	0.014845	0.014353
100.003	0.0122797	0.00163111	0.000746	0.030998	0.029985
149.999	0.0186143	0.00247022	0.001116	0.047118	0.045613
200.005	0.0248356	0.00329642	0.001474	0.063205	0.061239
250.004	0.0309054	0.00410499	0.00182	0.079292	0.076815
300.003	0.0367898	0.00488855	0.002153	0.09536	0.092401
350	0.0424625	0.00564808	0.002475	0.111432	0.107986
399.998	0.0477284	0.0063593	0.002774	0.126956	0.123036
450	0.0529906	0.00706867	0.003072	0.142972	0.138541
500	0.0580487	0.00775507	0.003357	0.158977	0.153977
550.004	0.0629169	0.00841898	0.003633	0.174953	0.16938
600.001	0.0676035	0.00906101	0.0039	0.190911	0.184728
650.002	0.072112	0.00968523	0.004157	0.206851	0.20005
700.001	0.0764515	0.0102889	0.004406	0.222754	0.215296
749.999	0.0806437	0.010872	0.004648	0.238712	0.230511
800	0.0846774	0.0114382	0.004881	0.254564	0.245674
849.999	0.0885798	0.0119888	0.005107	0.270451	0.260758
900.002	0.0923286	0.0125204	0.005326	0.286323	0.275813
949.998	0.0959611	0.0130374	0.00554	0.302151	0.290746
1000.01	0.0994722	0.013541	0.005747	0.317996	0.30567
2000	0.149194	0.0210258	0.008877	0.627722	0.58907
3000.01	0.17339	0.0250098	0.01062	0.920986	0.842481
4000.02	0.185008	0.0269956	0.011555	1.18885	1.06372
5000.01	0.190838	0.0279357	0.012036	1.41602	1.24547
6000	0.194091	0.0283459	0.012243	1.57324	1.36973
7000	0.195992	0.0284941	0.012272	1.63232	1.41411
8000	0.19726	0.0285543	0.012241	1.65287	1.42819
9000	0.198194	0.0285688	0.012194	1.66567	1.43672
10,000	0.198834	0.0285681	0.012135	1.67456	1.44254
11,000	0.199363	0.0285517	0.012067	1.68139	1.44686
12,000	0.19976	0.0285216	0.012	1.68652	1.45043
13,000	0.200064	0.028479	0.011925	1.69044	1.45294
14,000	0.20024	0.0284272	0.011848	1.69331	1.4546

**Table 2:** Raw data for the magnetic properties of samples with different microcarbonyl iron loading.

Field (G)	Moment/Mass (emu/g)			
	B0	B10	B20	B30
14,000	−0.031075	11.8639	22.0397	32.3863
13,000	−0.02804	11.8436	22.0025	32.3321
12,000	−0.0250311	11.8173	21.956	32.2627
11,000	−0.0219739	11.7842	21.8972	32.1692
10,000	−0.0189939	11.7385	21.8192	32.0514
9000	−0.0161259	11.6749	21.7153	31.8884
7999.99	−0.01318	11.5722	21.5555	31.6351
7000	−0.0102412	11.3408	21.2215	31.0778
5999.99	−0.00757066	10.5973	20.0837	29.3183
5000	−0.00487035	9.36743	18.0113	26.208
4000	−0.00231871	7.86957	15.3323	22.2357
2999.99	5.37453E-05	6.17344	12.174	17.6033
2000	0.00220753	4.29103	8.55808	12.3479
999.995	0.00375761	2.2261	4.48136	6.46013
950.001	0.00379638	2.11787	4.26479	6.14849
899.996	0.00381628	2.00971	4.04823	5.83543
850.002	0.0038642	1.90109	3.83049	5.5222
799.996	0.00387269	1.79226	3.61202	5.20668
749.998	0.00393742	1.68291	3.39264	4.89022
699.994	0.00393191	1.57331	3.17245	4.5725
649.998	0.00395945	1.46341	2.95114	4.25417
599.995	0.00391781	1.35319	2.72923	3.93412
549.997	0.00395484	1.24262	2.50665	3.61309
499.994	0.00392785	1.13188	2.28316	3.29095
449.995	0.00388682	1.02083	2.05926	2.96768
399.996	0.00383848	0.909494	1.83417	2.64356
349.992	0.00379398	0.799289	1.6114	2.3226
299.999	0.00368892	0.687711	1.38585	1.99754
249.998	0.00359657	0.575731	1.15962	1.67148
199.997	0.00342496	0.463652	0.933475	1.34533
149.994	0.00322757	0.351568	0.707091	1.01876
99.9997	0.00291722	0.239467	0.480419	0.692181
49.9982	0.00242662	0.127058	0.253698	0.3655
−0.00121525	0.00130904	0.014001	0.026346	0.038273
−49.9993	1.42115E-05	−0.09915	−0.20137	−0.2897
−100	−0.000706656	−0.21194	−0.42856	−0.61703
−150.004	−0.00123323	−0.32438	−0.65539	−0.94375
−200.001	−0.00163454	−0.43683	−0.88226	−1.27127
−250.005	−0.00195675	−0.54905	−1.10896	−1.59797
−300.001	−0.00222473	−0.66094	−1.33515	−1.9239
−350.001	−0.00242407	−0.77293	−1.56122	−2.24968
−400.003	−0.00261376	−0.88055	−1.77788	−2.56246
−450.001	−0.00279957	−0.99198	−2.00252	−2.88633
−500.002	−0.00290754	−1.10343	−2.22714	−3.20993
−550.001	−0.00298129	−1.21452	−2.45117	−3.53301
−600.002	−0.00305508	−1.32511	−2.67429	−3.85456
−649.997	−0.00309795	−1.43558	−2.89671	−4.17541
−700.004	−0.00312656	−1.54577	−3.11825	−4.495
−750.001	−0.00314179	−1.6557	−3.33933	−4.81319
−800.001	−0.00317582	−1.76512	−3.55872	−5.13013
−850.003	−0.00312649	−1.87406	−3.77758	−5.44496
−900.003	−0.00314159	−1.98258	−3.99525	−5.75866
−950.004	−0.00310596	−2.09074	−4.21211	−6.07099
−1000	−0.00310427	−2.1984	−4.42774	−6.38198
−2000.01	−0.00182224	−4.26783	−8.51634	−12.285
−3000.02	5.55426E-05	−6.15391	−12.1412	−17.5501
−4000.02	0.0022014	−7.85834	−15.3145	−22.2024
−5000.01	0.0045618	−9.35976	−18.0001	−26.184
−6000.01	0.0070027	−10.5869	−20.0691	−29.2915
−7000.01	0.00959641	−11.3387	−21.2209	−31.0663
−8000	0.0122909	−11.5689	−21.5515	−31.6209
−9000.01	0.0149946	−11.6698	−21.7087	−31.8705
−10,000	0.0177142	−11.7329	−21.8132	−32.0355
−11,000	0.0204891	−11.7783	−21.8891	−32.1547
−12,000	0.0232754	−11.8119	−21.9466	−32.2466
−13,000	0.0261698	−11.8371	−21.9937	−32.3154
−13,998.8	0.028921	−11.8562	−22.0284	−32.3694
−13,000	0.025876	−11.837	−21.9922	−32.3172
−12,000	0.0231271	−11.8111	−21.9476	−32.2423
−11,000	0.0203238	−11.7767	−21.8892	−32.1557
−10,000	0.0176286	−11.7313	−21.8131	−32.0355
−9000	0.0148564	−11.6673	−21.7073	−31.8706
−8000	0.0122396	−11.5658	−21.5492	−31.6225
−7000	0.00942324	−11.3378	−21.2198	−31.0688
−6000	0.0067575	−10.5914	−20.076	−29.3052
−4999.99	0.00424855	−9.36889	−18.0163	−26.2126
−4000	0.00185859	−7.87186	−15.3392	−22.2428
−3000	−0.000454371	−6.17206	−12.1748	−17.6022
−2000	−0.00245206	−4.29079	−8.56019	−12.3491
−999.991	−0.00386748	−2.22359	−4.47747	−6.45341
−950.002	−0.0038733	−2.11529	−4.26109	−6.14091
−899.996	−0.00391246	−2.00716	−4.04434	−5.82871
−849.999	−0.00396035	−1.89857	−3.82672	−5.51487
−799.999	−0.00396012	−1.78975	−3.60803	−5.20009
−750.003	−0.00398328	−1.68042	−3.38871	−4.88345
−699.997	−0.00399258	−1.57054	−3.16771	−4.56517
−649.994	−0.00399586	−1.4604	−2.94627	−4.24589
−599.996	−0.00401965	−1.34997	−2.72383	−3.92551
−549.994	−0.00400039	−1.23938	−2.50073	−3.60406
−500	−0.00396898	−1.12836	−2.2766	−3.28081
−449.998	−0.00394523	−1.01711	−2.05218	−2.95728
−400	−0.00383896	−0.90586	−1.82726	−2.633
−349.99	−0.0038138	−0.79783	−1.60962	−2.31856
−299.994	−0.00372182	−0.6866	−1.38492	−1.99457
−249.998	−0.00361214	−0.57451	−1.15869	−1.6684
−199.996	−0.00341677	−0.46235	−0.93179	−1.34103
−149.998	−0.0032138	−0.35002	−0.70491	−1.0141
−99.9965	−0.00291814	−0.23765	−0.47793	−0.6869
−50.0004	−0.00237836	−0.12508	−0.251	−0.35939
−0.00056425	−0.00126111	−0.01213	−0.02336	−0.03209
50.0035	−5.95938E-06	0.101123	0.204394	0.295678
100.004	0.0007895	0.213759	0.431211	0.622203
149.997	0.00130243	0.326092	0.657792	0.948996
200.004	0.001702	0.438233	0.884208	1.2755
250.002	0.00202766	0.550379	1.11039	1.60166
300.003	0.00227562	0.662328	1.33671	1.92756
350.002	0.00253152	0.774245	1.56278	2.25309
399.997	0.00270354	0.884308	1.78525	2.57347
450.001	0.00284257	0.995711	2.01022	2.89753
500.001	0.00296913	1.10681	2.2343	3.22063
549.999	0.00306319	1.21779	2.45766	3.54278
599.998	0.00314035	1.32826	2.68047	3.86393
650.003	0.00318115	1.43852	2.90276	4.1842
700.001	0.0032219	1.54855	3.12379	4.50276
749.998	0.00325021	1.65826	3.34435	4.82039
800	0.0032365	1.76741	3.56395	5.13698
850.001	0.00324271	1.87625	3.78253	5.45198
899.997	0.00324259	1.98481	4.00004	5.76547
950.003	0.00321438	2.0929	4.21675	6.07802
999.999	0.00321141	2.20071	4.43249	6.38897
2000	0.00201758	4.26672	8.5146	12.281
3000.01	0.000164184	6.1532	12.1402	17.5473
4000	−0.00187505	7.85265	15.3054	22.1885
5000.01	−0.00415965	9.35352	17.9908	26.1701
6000	−0.00658414	10.5862	20.0695	29.2913
7000	−0.00910688	11.3329	21.2144	31.055
8000	−0.0117834	11.5645	21.5483	31.6142
9000	−0.0144309	11.6668	21.7061	31.8651
10,000	−0.0171603	11.7289	21.8086	32.0271
11,000	−0.0198124	11.7743	21.8859	32.1447
12,000	−0.0226373	11.8085	21.9442	32.2371
13,000	−0.0253815	11.8338	21.9911	32.3091
14,000	−0.0281689	11.8532	22.0258	32.3622

## 3 Experimental Design, Materials and Methods

### 3.1 Materials

**Fig. 1:**
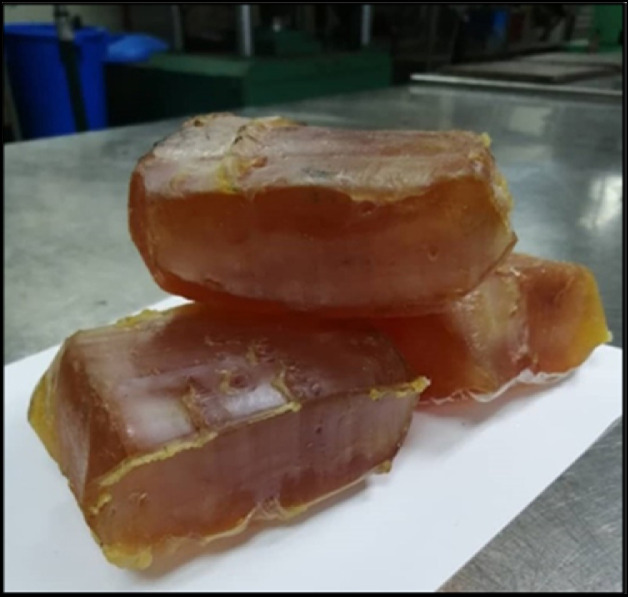
Natural rubber (Grade SMR L).

**Fig. 2:**
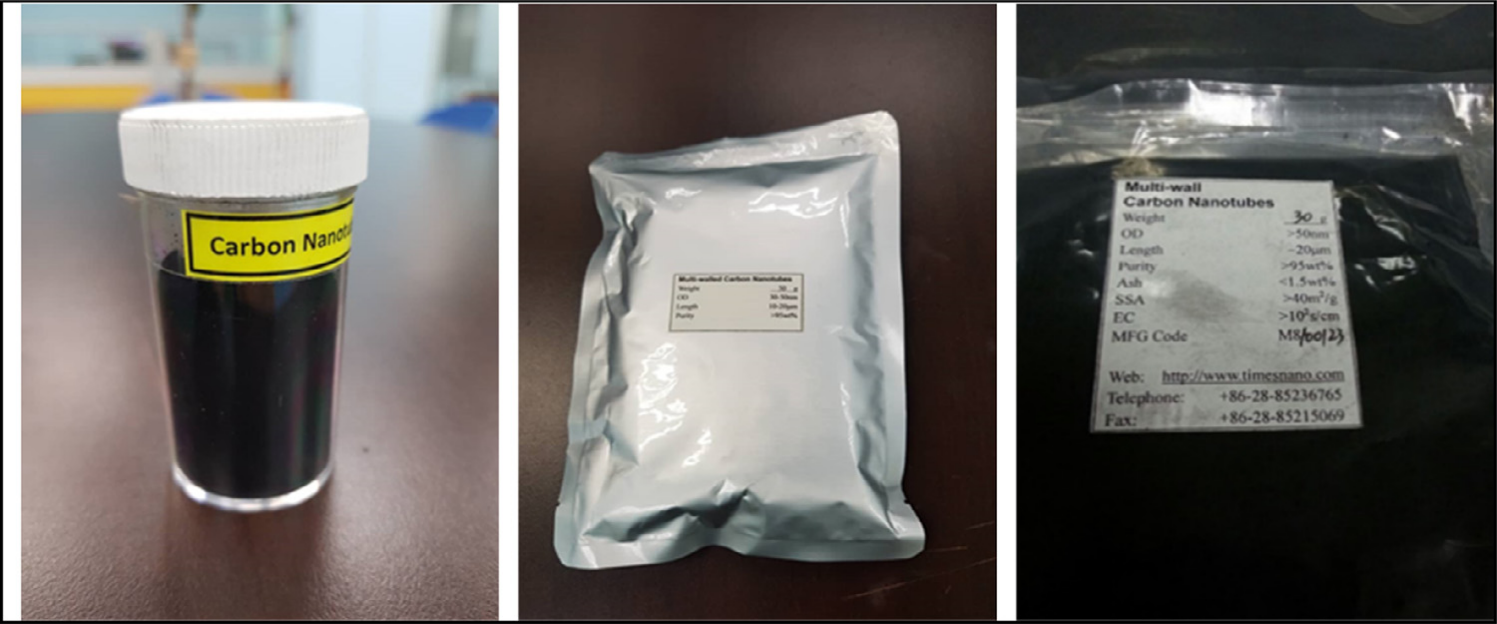
Multi-walled carbon nanotube (MWCNT).

**Fig. 3:**
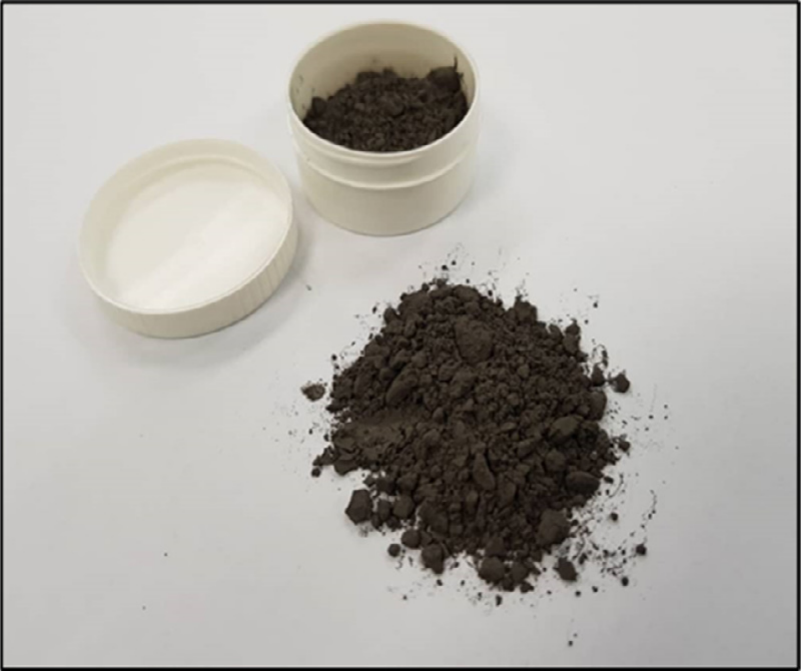
Microcarbonyl iron powder.

Elastic matrix and magnetic particles are the main ingredients of nanocomposites elastomer. In this experiment study, Standard Malaysian Rubber (SMR) L grade natural rubber was chosen as matrix-based nanocomposites natural rubber compound as shown in Fig. 1. Fig. 2 shows the carbo nanotubes filler was purchased from Chengdo Organic Chemical Co. Ltd., Chinese Academy of Sciences. In order to develop the nanocomposites natural rubber compounds, carbonyl iron particles with The diameter and density of the iron particle are in range of 6 to 9 µm and 7.86 g/cm^3^, respectively were purchased from Sigma-Aldrich Sdn. Bhd. (M) (Fig. 3).

**Fig. 4:**
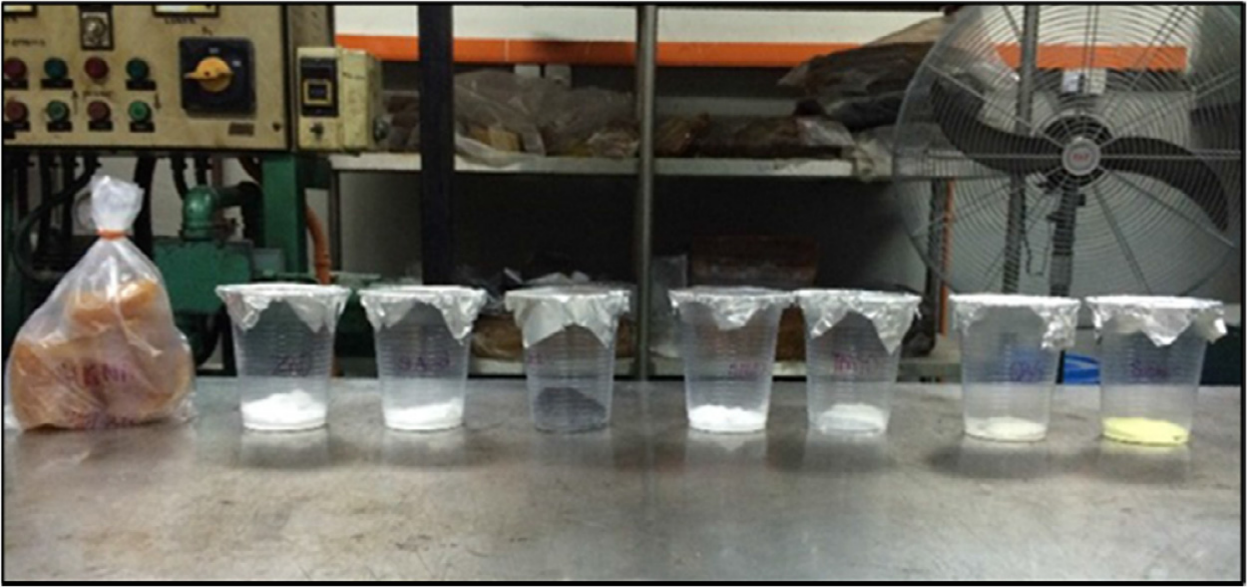
Rubber compounding components.

Carbon black N220 was used as the reinforcing filler of the nanocomposites elastomer compound. Other materials such as zinc oxide (ZnO), stearic acid and sulphur are also required as the basic ingredients of compounding unfilled rubber or filled rubber. In rubber standard compounds, (ZnO) and stearic acid have been used as activator and co-activator respectively. Cyclohexyl benzothiazolesulfenamide (CBS) and tetramethylthiuram disulphide (TMTD) are the accelerator and additives that had been selected to increase the properties of elastomers. Besides that, they were added as to help the vulcanization system. Fig. 4. Shows the rubber compounding components.

### 3.2 Experimental Design and Methods

**Fig. 5:**
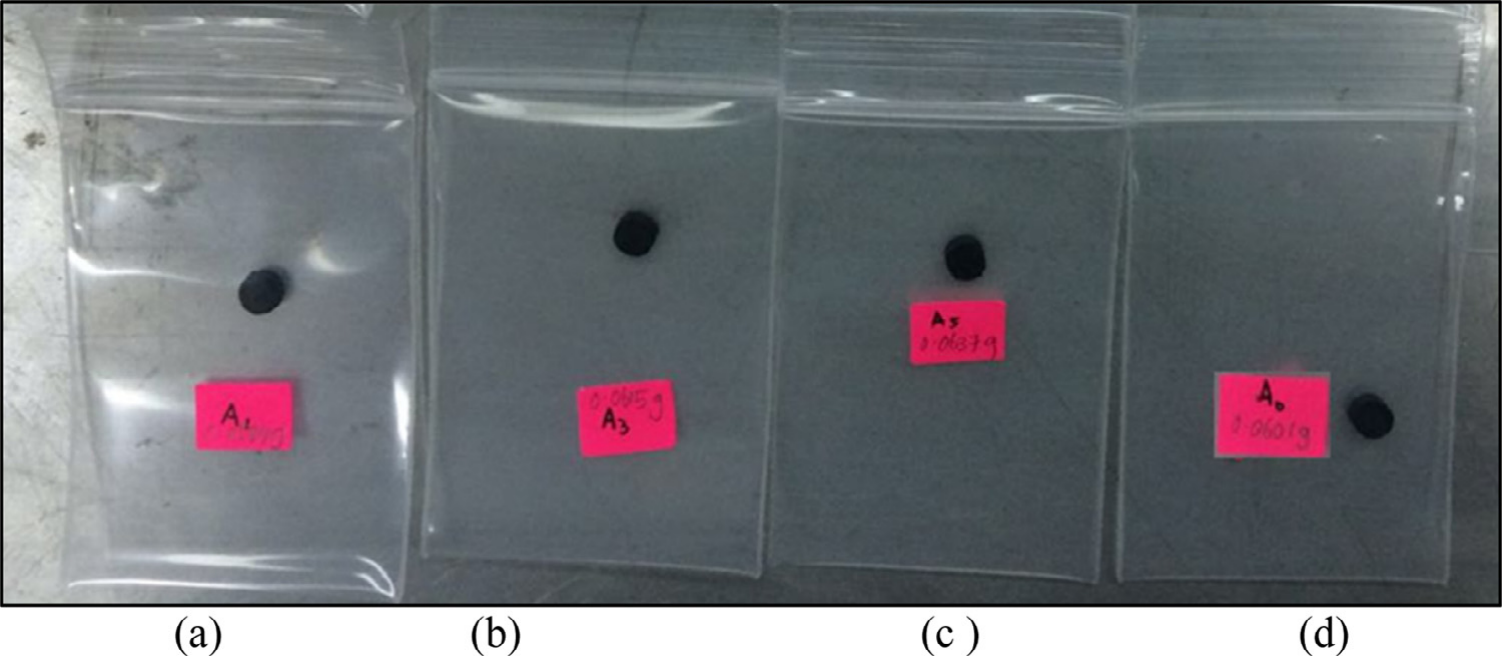
Magnetic test specimens for different nanocarbon content (for batch A): (a) 1%, (b) 3%, (c) 5%, and (d) 0%.

**Fig. 6:**
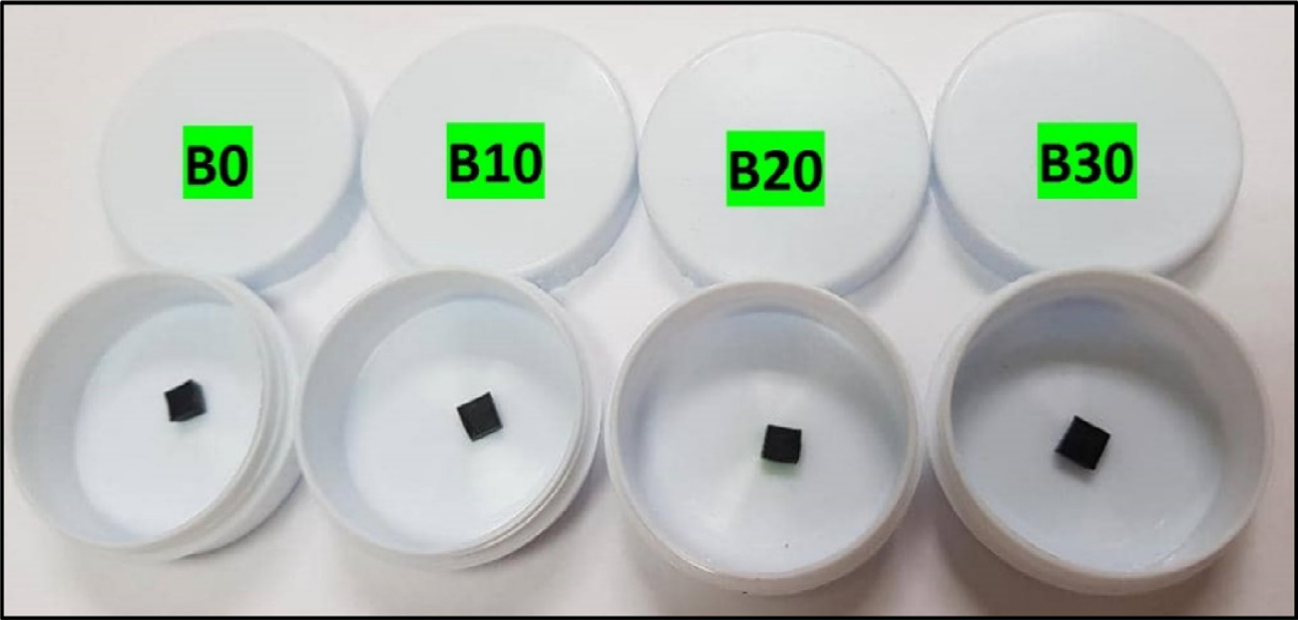
Magnetic test specimens for different microcarbonyl iron content (for batch B): (a) 0%, (b) 10%, (c) 20%, and (d) 30%.

There are two batches of sample as shown in Fig. 5 for Batch A with different nanocarbon content and Fig. 6 for Batch B with different microcarbonyl iron content. Before conducting the test, the weight of each sample was determined in gram by an analytical balance. A small amount of high vacuum grease was used to attach the samples to the tube holder. Then, the tube holder was inserted in the VSM. The input data such as weight of the samples and the magnetic moment were recorded in the computer system. Thus, the test was run until the result obtained. For comparison, pure microcarbonyl iron was checked in the powder state. The microcarbonyl iron was sealed with the transparent adhesive tape on both sides before being tested to ensure fine powder adheres throughout the test. The compounding process of a batch mass of nanocomposites elastomer was made by following BS ISO 2393 [1]. The rubber compounds were obtained in sheets and conditioned at 23± °C for 24 h before cure assessment. The compounding process of nanocomposites elastomer development was done using two roll mills and a conventional vulcanization system. The cure assessment of nanocomposites elastomer was determined by Rheometer 100. The temperature was set at 150 °C for each sample.

For the material to be used for civil engineering applications, the nanocomposites elastomer compound should satisfy and achieve the following general performances and quality control requirements according to BS ISO 6446 [2].

The performance of magnetic rubber device is dependent on mechanical and magnetic properties of composite rubber materials. A vibrating sample magnetometer (VSM) is an instrument to detect the magnetic properties.

**Fig. 7:**
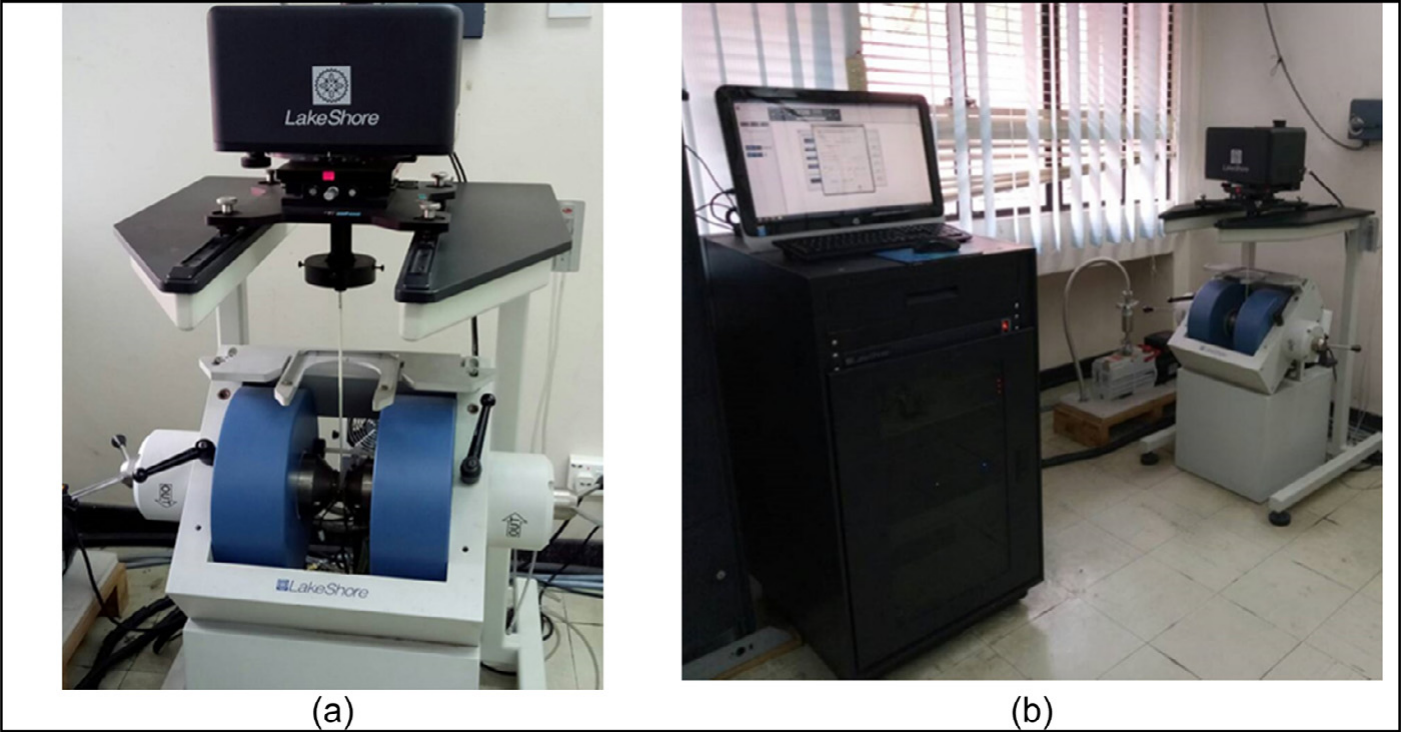
VSM lakeshore instrument.

The magnetic field versus magnetization was carried out to study the magnetic characteristics of the nanocomposite samples. VSM Lakeshore 7404 Series was used to analysis the magnetic properties of nanocomposite material as shown in Fig. 7.

#### 3.2.1 Variation of Nanocarbon Loading

**Fig. 8:**
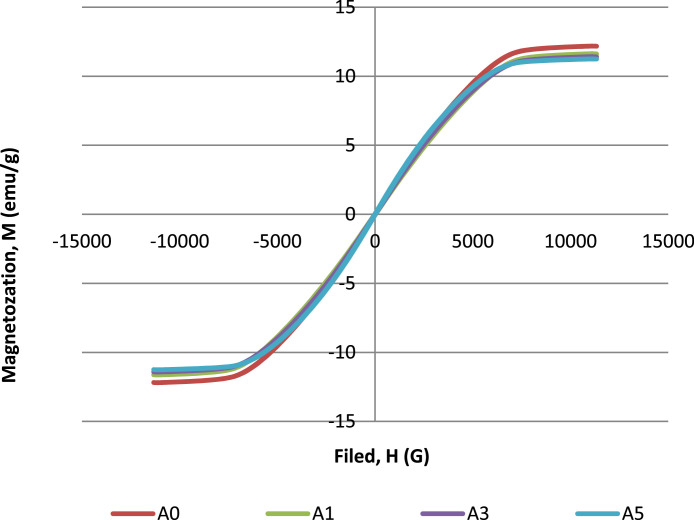
Hysterisis loops with different nanocarbon loading.

At first, the pure magnetic filler, microcarbonyl iron (MCI) was checked in the powder state. The magnetic value of microcarbonyl iron has a value of 84.017 emu/g, which is supposedly greater than the composites. A0 is the control sample where to identify the effect of nanocarbon in the presence of microcarbonyl iron in the composite. The raw data on the magnetic properties of composites with different nanocarbon loading are tabulated in Table 1. Fig. 8 shows the hysteresis loops for NCE composites with the different nanocarbon loading.

#### 3.2.2 Variation of Microcarbonyl Iron Loading

**Fig. 9:**
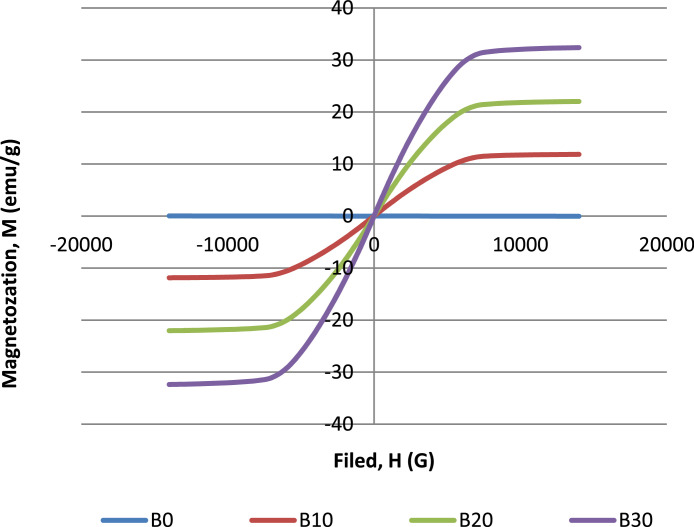
Hysterisis loops with different microcarbonyl iron loading.

In variation of microcarbonyl iron loading, all samples were expected to show their magnetic properties. B0 is the control sample where to identify the presence of magnetic properties in the composite. The optimum amount of 1 pphr CNTs in specimens are fabricated with different microcarbonyl iron loadings: 0 pphr (B0), 10 pphr (B10), 20 pphr (B20), and 30 pphr (B30). Table 2 shows the data with different microcarbonyl iron loading with magnetic strength is about 14,000 G. Fig. 9 shows the hysterisis loops with different microcarbonyl iron loading with magnetic strength is about 14,000 G.

## Ethics Statements

Here confirming that our work involved data collected from laboratory work. The experiments complied with the University guidelines and were carried out in accordance with an associated standard as guideline.

## CRediT authorship contribution statement

**Rozaina Ismail:** Conceptualization, Methodology, Investigation, Data curation, Writing – original draft, Writing – review & editing, Visualization. **Azmi Ibrahim:** Supervision, Writing – review & editing. **Hamidah Mohd.Saman@Hj. Mohamed:** Supervision, Writing – review & editing. **Mohamad Rusop Mahmood:** Supervision, Writing – review & editing. **Azlan Adnan:** Supervision, Writing – review & editing.

## Declaration of Competing Interest

The authors declare that they have no known competing financial interests or personal relationships that could have appeared to influence the work reported in this paper.

## Data Availability

Raw Data of Magnetic Test for Variation of Microcarbonyl iron of Loading Natural Rubber Nanocomposites (Original data) (Mendeley Data).
